# The impact of peroxisome proliferator‐activated receptor‐γ activating angiotensin receptor blocker on outcomes of patients receiving immunotherapy

**DOI:** 10.1002/cam4.5734

**Published:** 2023-02-24

**Authors:** Cho‐Han Chiang, Yu‐Cheng Chang, Shih‐Syuan Wang, Yuan‐Jen Chen, Xin Ya See, Chun‐Yu Peng, Yuan Ping Hsia, Cho‐Hsien Chiang, Cho‐Hung Chiang, Cheng‐Ming Peng

**Affiliations:** ^1^ Department of Medicine Mount Auburn Hospital, Harvard Medical School Boston Massachusetts USA; ^2^ Da Vinci Minimally Invasive Surgery Center Chung Shan Medical University Hospital Taichung Taiwan; ^3^ Department of Medicine Taipei Veterans General Hospital Taipei Taiwan; ^4^ Department of Medicine Unity Hospital, Rochester Regional Health Rochester New York USA; ^5^ Department of Medicine Danbury Hospital Danbury Connecticut USA; ^6^ Department of Family Medicine Taipei Tzu Chi Hospital, Buddhist Tzu Chi Medical Foundation New Taipei City Taiwan; ^7^ Department of Medical Education Kuang Tien General Hospital Taichung Taiwan; ^8^ London School of Hygiene & Tropical Medicine London UK; ^9^ Department of Internal Medicine National Taiwan University Hospital Taipei Taiwan; ^10^ Department of General Division Taipei Tzu Chi Hospital, Buddhist Tzu Chi Medical Foundation New Taipei City Taiwan; ^11^ School of Medicine Chung Shan Medical University Taichung Taiwan

**Keywords:** angiotensin receptor blockers, immune checkpoint inhibitors, peroxisome proliferator‐activated receptor‐γ, renin‐angiotensin‐aldosterone system inhibitors

## Abstract

**Background:**

Certain angiotensin receptor blockers (ARBs) have peroxisome proliferator‐activated receptor‐γ (PPAR‐γ) activation property, which has been associated with improved programmed cell death ligand 1 blockade and cytotoxic T lymphocyte‐mediated antitumor activity.

**Methods:**

We conducted a retrospective cohort study to investigate the impact of PPAR‐γ‐activating ARBs on patient survival in patients treated with immune checkpoint inhibitors (ICIs) across all types of cancers.

**Results:**

A total of 167 patients receiving both angiotensin receptor blockers (ARBs) and immune checkpoint inhibitors (ICIs) were included. Compared with non‐PPAR‐γ‐ARB users (*n* = 102), PPAR‐γ‐ARB users (*n* = 65) had a longer median overall survival (not reached [IQR, 16.0—not reached] vs. 18.6 [IQR, 6.1–38.6] months) and progression‐free survival (17.3 [IQR, 5.1—not reached] vs. 8.2 [IQR, 2.4–18.6] months). In Cox regression analysis, the use of PPAR‐γ‐activating ARBs had an approximately 50% reduction in all‐cause mortality and disease progression. Patients who received PPAR‐γ‐activating ARBs also had higher clinical benefit rates than non‐PPAR‐γ‐ARB users (82% vs. 61%, *p* = 0.005).

**Conclusion:**

The use of ARBs with PPAR‐γ‐activating property is linked with better survival among patients receiving ICIs.

## Graphical abstract

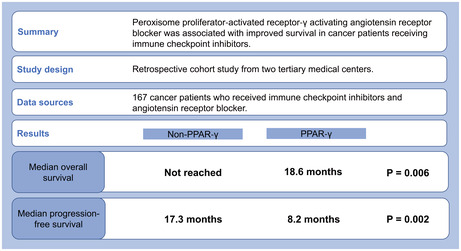

## 1 INTRODUCTION

Over the past decade, immune checkpoint inhibitors (ICIs) have offered a promising and effective treatment modality for advanced cancers.^1^ Around 40% of cancer patients were estimated to be eligible for ICI treatment; however, only <20% of these patients respond to ICIs.^1^ It is therefore imperative to develop strategies to augment and improve the response to ICI treatment. In addition to novel drug discovery, repurposing existing drugs for new indications has become a cost‐effective strategy that can potentially improve the survival outcomes of cancer patients.^2^, ^3^, ^4^, ^5^, ^6^, ^7^

Accumulating evidence from previous studies showed a trend of better survival outcomes in ICI patients who were concomitantly treated with certain medications, such as renin‐angiotensin‐aldosterone system inhibitors (RAASi).^3^, ^7^, ^8^, ^9^, ^10^, ^11^ Experimental studies have shown that the renin‐angiotensin‐aldosterone system (RAAS) plays an important role in regulating the immune response against tumor cells.^12^, ^13^ Consistent with preclinical data, clinical studies showed that patients who used ICI and RAASi had a higher rate of anti‐tumor effect survival than those who did not use RAASi.^7^, ^8^, ^10^, ^11^, ^14^

RAASi are composed of two classes of medications, angiotensin receptor blockers (ARBs) and angiotensin‐converting enzyme inhibitors (ACEIs). Both ARBs and ACEIs were linked with improved survival in patients treated with immunotherapy. However, it is currently unknown which classes of RAASi are associated with improved patient outcomes. Furthermore, it is unclear how the biochemical properties or structures of each RAASi correlate with improved treatment response. Of note, certain ARBs such as telmisartan, irbesartan, losartan, and candesartan are known as peroxisome proliferator‐activated receptor‐γ (PPAR‐γ) activators.^15^, ^16^, ^17^, ^18^, ^19^ PPAR‐γ activation has been reported to increase programmed cell death ligand 1 (PD‐1) blockade and cytotoxic T lymphocyte‐mediated antitumor activity.^20^, ^21^ Based on these preclinical data, we hypothesized that patients treated by ICIs who were concomitantly placed on ARBs with PPAR‐γ activating property would have a superior survival outcome than patients who were placed on non‐PPAR‐γ activating ARBs. The aim of this study was to evaluate the impact of PPAR‐γ activating ARBs on survival benefits in patients receiving ICIs.

## 2 METHODS

This retrospective cohort study was carried out at two tertiary referral centers in Taiwan. This study was approved by the Institutional Review Boards (IRB) at both hospitals.^22^ We included patients who were administered at least 2 cycles of immunotherapy between 2015 and 2021. We excluded participants with incomplete data, non‐adult patients, and those who only received 1 cycle of immunotherapy. We collected information on patient demographics and clinicopathological characteristics, including age, sex, Eastern Cooperative Oncology Group (ECOG) Performance Status (ECOG‐PS), cancer stage, cancer pathology, ICI class, treatment cycle, and underlying comorbidities. The classification of ICIs was presented in Table S1. We also collected data regarding the type of angiotensin II receptor blockers (ARBs) according to PPAR‐γ properties. PPAR‐γ‐activating ARBs include telmisartan, irbesartan, candesartan, and losartan, while non‐PPAR‐γ‐activating ARBs include olmesartan and valsartan (Table S2).^15^, ^16^, ^17^, ^18^, ^19^ We defined PPAR‐γ‐users as patients using PPAR‐γ‐activating ARBs after ICI initiation and non‐PPAR‐γ‐ARB users as patients using non‐PPAR‐γ‐activating ARBs after ICI initiation. We set the primary endpoint as overall survival (OS) and the secondary endpoints as progression‐free survival (PFS) and clinical benefit rates. Patients were considered to have achieved clinical benefits if they had stable disease, partial remission, or complete remission of the tumor. The tumor‐based endpoints were determined by clinician evaluation and radiological imaging using a previously validated approach.^23^

### 2.1 Statistical analysis

We used the Kaplan–Meier analysis and Cox proportional hazard model analysis to assess the OS and PFS between PPAR‐γ users and non‐users. For multivariate Cox models, we selected variables a priori and these include age, gender, performance status, cancer stage, cancer type, hypertension, hyperlipidemia, diabetes mellitus, ischemic heart disease, chronic kidney disease, arrhythmia, heart failure, surgery, concomitant use of cardiovascular medications, ICI class, combination ICI therapy, and cancer treatment. We compared the clinical benefit rates between PPAR‐γ users and non‐users using the chi‐squared test. We conducted a subgroup analysis for patients with lung cancer. The other cancer types were unable to be analyzed due to their small sample sizes. We set a *p*‐value of less than 0.05 as being statistically significant. We performed all analyses with Stata version 16.0 (StataCorp LLC.).

## 3 RESULTS

There were a total of 878 patients who received ICIs in our study. After excluding patients who had incomplete data, who received only 1 cycle of ICI, or non‐adult patients, there remained 65 PPAR‐γ‐users and 102 non‐users in this study (Figure S1). The median age for PPAR‐γ‐users and non‐PPAR‐γ‐users is 67 (59–74) and 65 (58–72) years old, respectively (Table S2). For both groups, lung cancer (56%) and hepatobiliary cancer (19%) were the most common cancers. The patient demographics were summarized in Table S3.

### 3.1 Outcomes

**FIGURE 1:**
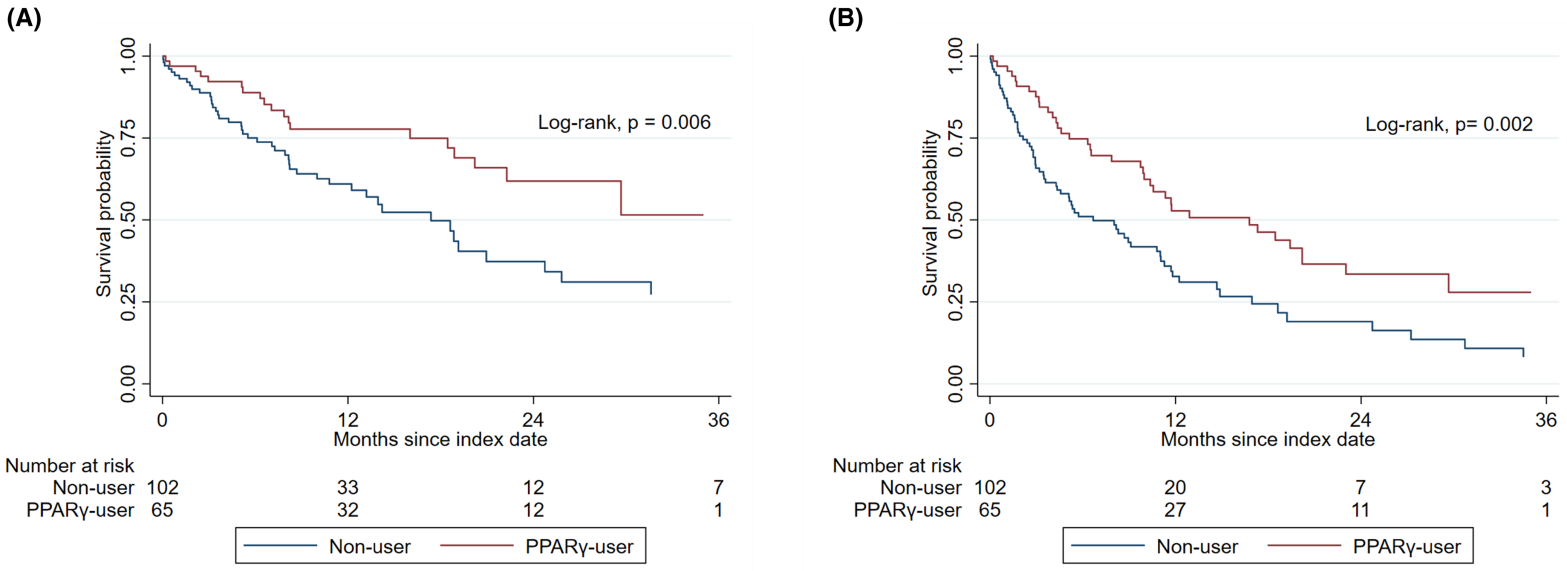
Overall and progression‐free survival of patients treated with and without PPAR‐y‐activating ARBs. (A) Overall survival. (B) Progression‐free survival. ARBs, angiotensin receptor blockers; PPAR‐y, peroxisome proliferator‐activated receptor‐γ.

Compared with non‐users, PPAR‐γ‐users had a longer median OS (not reached [IQR, 16.0—not reached] vs. 18.6 [IQR, 6.1–38.6] months; *p*‐value = 0.006) and PFS (17.3 [IQR, 5.1—not reached] vs. 8.2 [IQR, 2.4–18.6] months; *p*‐value = 0.002) (Figure 1). Using a univariate Cox regression analysis, the use of PPAR‐γ‐activating ARBs had an approximately 50% reduction in all‐cause mortality and disease progression compared to non‐users (Table 1). In a multivariate Cox regression model, a similar risk reduction was also observed after adjusting for underlying comorbidities and cancer therapy (Table 1). Consistent with these findings, the use of PPAR‐γ‐activating ARBs was associated with higher clinical benefit rates when compared with non‐users (82% vs. 61%, *p* = 0.005) (Table S4).

**TABLE 1:** Cox proportional hazard analysis of use of PPAR‐γ and all‐cause mortality and disease progression.

Analysis	Outcome	Univariate HR (95% IC)	*p*‐value	Multivariate HR (95% IC)^a^	*p* value
PPARγ‐user versus Non‐user	All‐cause mortality	0.47 (0.27–0.82)	0.007	0.48 (0.24–0.95)	0.035
	Disease progression or mortality	0.53 (0.35–0.79)	0.002	0.49 (0.30–0.81)	0.005

Abbreviation: PPAR‐y, peroxisome proliferator‐activated receptor‐γ.

^a^ Multivariate analysis includes variables: age, sex, cancer type, cancer stage, Eastern Cooperative Oncology Group Performance Status, surgery, underlying comorbidities such as hypertension, diabetes mellitus, chronic kidney disease, heart failure, ischemic heart disease, arrhythmia, hyperlipidemia, the use of medications such as beta‐blocker, calcium channel blocker, statin, aspirin, cancer treatment including VEGF inhibitor, taxanes, vinca alkaloids, platinums, tyrosine kinase inhibitors, anthracyclines alkylating agents, topoisomerase‐1 inhibitors, antimetabolites, ICI class including PD‐1 inhibitors, PD‐L1 inhibitors, CTLA‐4 inhibitors, and combination immune checkpoint inhibitor therapy.

### 3.2 Subgroup analysis

Among 167 patients in the study, 93 (56%) were patients with lung cancer, of which 46 and 47 patients were treated with non‐PPAR‐γ activating and PPAR‐γ activating ARBs, respectively. In the univariate Cox regression analysis, lung cancer patients treated with PPAR‐γ activating ARBs had an approximately 60% reduction in all‐cause mortality and a 50% reduction in disease progression rate (Table S5). In the multivariate Cox regression analysis, the use of PPAR‐γ activating ARBs remained statistically significantly associated with a reduction in all‐cause mortality and disease progression after adjusting for potential confounders including age, sex, and cancer stage (Table S5).

## 4 DISCUSSION

In this cohort study, ICI‐treated patients who concurrently received PPAR‐γ activating ARBs had lower all‐cause mortality and tumor progression rates than those who received non‐PPAR‐γ activating ARBs. These findings add to the current literature on the possible survival benefits of RAASi associated with immunotherapy. Based on a previous observational study, Jain et al. reported that the concomitant use of RAASi had a higher rate of tumor response in metastastic urothelial cell carcinoma.^14^ Another large study showed that RAASi was linked with survival benefits in hypertensive patients receiving ICI treatment.^8^

It is currently unclear how the PPAR‐γ activating property of certain ARBs led to improved response to immunotherapy. ARBs are RAASi that work primarily as antagonists on the angiotensin II receptors.^24^ Certain ARBs, such as telmisartan, irbesartan, losartan, and candesartan possess PPAR‐γ activating properties that are tightly associated with the expression of immune checkpoint regulators.^15^, ^16^, ^17^, ^18^, ^19^ For example, PPAR‐γ agonists have been shown to induce programmed death‐ligand 1 (PD‐L1) expression in colorectal cancer cell lines^25^; activation of PPAR‐γ also promotes programmed cell death protein 1 (PD‐1) expression in innate lymphoid cells.^26^ Taken together, PPAR‐γ activating ARBs may improve the response to ICIs synergistically. Furthermore, PPAR‐γ activation is associated with anti‐tumor effects via mechanisms other than immune checkpoint regulation. In lung cancer models, the activation of PPAR‐γ has been shown to reduce the invasiveness of tumor cells through the blockade of the MAPK pathway.^27^ In hepatocellular carcinoma (HCC) models, PPAR‐γ activation has been shown to block the proliferation of HCC cells by blocking the receptor for advanced glycation end product signaling.^28^ Coincidentally, lung and hepatobiliary cancer were two of the most predominant cancer types in our study population and might partially explain why we observe such substantial survival benefits. Conversely, PPAR‐γ inhibition, but not activation, was associated with improving ICI treatment in melanoma and breast cancer murine models.^29^, ^30^ Our subgroup analysis for lung cancer patients was consistent with the preclinical studies for lung cancer which showed improvement in both PFS and OS.^31^ Unfortunately, the sample size was small for other cancer types and subgroup analyses were not feasible.

Patients who received PPAR‐γ‐Activating ARBs had a lower rate of diabetes mellitus and higher rate of treatment with vascular endothelial growth factor (VEGF) inhibitors. It is important to note that diabetes mellitus is associated with a poorer prognosis in cancer patients, and may therefore confound the results by predisposing the non‐PPAR‐γ‐Activating ARBs group to a poorer prognosis.^32^ On the other hand, the impact of VEGF inhibitors on patient survival is more difficult to interpret. VEGF inhibitors were known to have a synergistic effect when used with ICIs.^33^ However, in a study conducted by Tanimura et al. the use of VEGF inhibitors prior to ICI initiation was associated with a poorer overall response rate.^34^ Of note, the use of PD‐L1 inhibitors was higher in the PPAR‐γ‐Activating ARB group, which may impact the OS and PFS associated with PPAR‐γ‐Activating ARB. We have included all of these variables in the multivariate Cox proportional models and found that the use of PPAR‐γ‐activating ARBs remains associated with improved patient survival even after adjusting for both of these covariates.

There are some limitations to this study. First, the retrospective nature of this study prevented us from collecting the tumor‐based endpoints using the Response Evaluation Criteria in Solid Tumors (RECIST).^23^ Nevertheless, we employed a validated approach that is more feasible in real‐world datasets. Second, the variables we have adjusted for multivariate Cox regression were not exhaustive and potential confounders such as line of therapy and sites of metastasis might still exist. Nevertheless, we have included a comprehensive list of confounders including cancer stage and prior surgery in the regression models. Furthermore, it is unlikely that the PPAR‐γ‐activating properties of ARBs influence how these medications were prescribed, and therefore these two groups of patients are likely to have similar baseline characteristics. Third, there was a considerable loss of follow‐up, which might have impacted the estimation of disease progression or mortality. Finally, the patient population of this study was mainly composed of Asian ethnicity, as a result, it is not certain whether the result can be extrapolated to other populations.

In conclusion, the concomitant use of ARBs with PPAR‐γ‐activating properties is associated with better survival outcomes and lower disease progression rates in patients receiving ICIs. Prospective clinical studies are required for further elucidation of the role of using PPAR‐γ‐activating ARBs with ICI therapy.

## AUTHOR CONTRIBUTIONS

**Cho Han Chiang:** Conceptualization (equal); data curation (equal); formal analysis (equal); investigation (equal); methodology (equal); project administration (equal); writing – original draft (equal); writing – review and editing (equal). **Yu‐Cheng Chang:** Formal analysis (equal); software (equal); validation (equal); writing – original draft (equal); writing – review and editing (equal). **Shih‐Syuan Wang:** Data curation (equal); formal analysis (equal); project administration (equal); validation (equal); visualization (equal). **Yuan‐Jen Chen:** Formal analysis (equal); investigation (equal); methodology (equal); software (equal); validation (equal); visualization (equal). **Xin‐Ya See:** Conceptualization (equal); data curation (equal); formal analysis (equal); validation (equal); visualization (equal). **Chun‐Yu Peng:** Investigation (equal); project administration (equal); validation (equal); visualization (equal). **Yuan Ping Hsia:** Conceptualization (equal); data curation (equal); formal analysis (equal); validation (equal); visualization (equal). **Cho Hsien Chiang:** Conceptualization (equal); data curation (equal); resources (equal); validation (equal); writing – review and editing (equal). **Cho Hung Chiang:** Data curation (equal); formal analysis (equal); methodology (equal); project administration (equal); supervision (equal); writing – original draft (equal); writing – review and editing (equal). **Cheng‐Ming Peng:** Investigation (equal); project administration (equal); supervision (equal); validation (equal).

## FUNDING INFORMATION

No funding.

## CONFLICT OF INTEREST STATEMENT

The authors have no conflict of interest to declare.

## ETHICS STATEMENT

The Institutional Review Board at Chung Shan Medical University Hospital and Taipei Tzu Chi Hospital approved this study (Chung Shan Medical University Hospital, number CS2‐21095; Taipei Tzu Chi Hospital, number 11‐X‐035). The Institutional review board at both hospitals waived informed consent.

## Supporting information


Data S1.

## Data Availability

Supporting data can be obtained on request from the corresponding author. Due to ethical considerations, these data are not publicly available.
