# Supplementary material for: The impact of peroxisome proliferator‐activated receptor‐γ activating angiotensin receptor blocker on outcomes of patients receiving immunotherapy
Source: Cancer Med. 2023 Feb 24;12(8):9583–8. doi: 10.1002/cam4.5734 (PMC10166924; doi:10.1002/cam4.5734)
Supplement: Supplementary file 1 — Data S1. [file CAM4-12-9583-s001.docx]

Supplemental Figure 1. Patient enrollment process


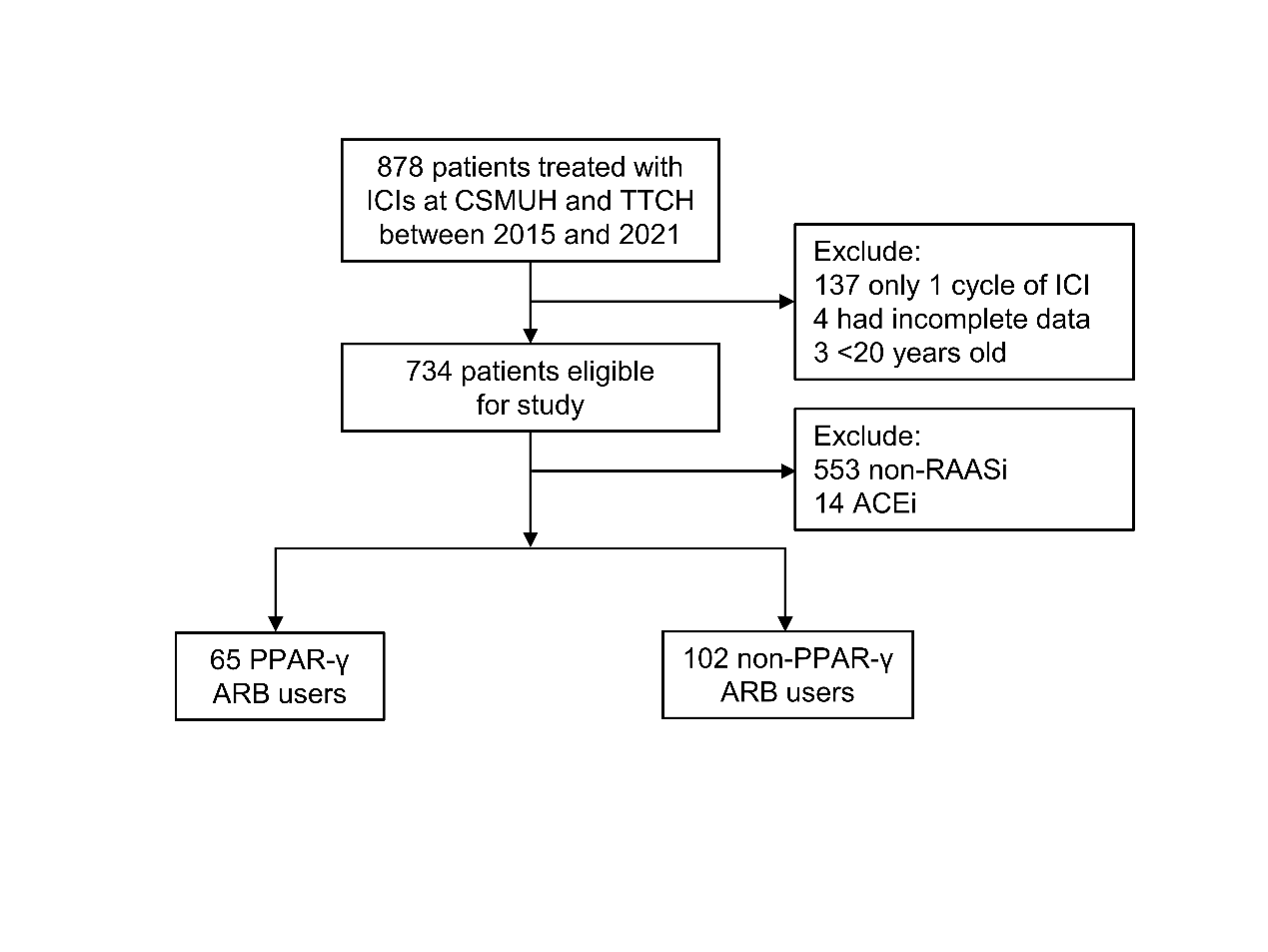


Abbreviations: ACEi, Angiotensin-converting enzyme inhibitor; ARB, angiotensin receptor blocker; CSMUH, Chung Shan Medical University Hospital; PPAR-γ, peroxisome proliferator-activated receptor-γ; ICI, immune checkpoint inhibitor; Renin-angiotensin-aldosterone system inhibitor; TTCH, Taipei Tzu Chi Hospital

Supplemental Table 1. Types of immune checkpoint inhibitors

| PD-1 inhibitor | PD-L1 inhibitor | CTLA-4 inhibitor |
| --- | --- | --- |
| Nivolumab  Pembrolizumab | Atezolizumab  Durvalumab | Ipilimumab |

Supplemental table 2. Breakdown of angiotensin receptor blocker

| PPAR-γ-activating ARB | Non-PPAR-γ-activating ARB |
| --- | --- |
| Irbesartan  Losartan  Telmisartan  Candesartan | Olmesartan  Valsartan |

Abbreviations: ARB, angiotensin receptor blocker; PPAR-γ, peroxisome proliferator-activated receptor-γ

Supplemental table 3. Patient demographics

|  | Total | Non-user | PPAR-γ user | P-value |
| --- | --- | --- | --- | --- |
|  | N=167 | N=102 | N=65 |  |
| Age | 66 (58-72) | 65 (58-72) | 67 (59-74) | 0.38 |
| Male | 103 (62%) | 71 (70%) | 32 (49%) | 0.008 |
| ECOG-PS **≥** 3 | 2 (1%) | 0 (0%) | 2 (3%) | 0.075 |
| Stage 4 | 122 (73%) | 76 (75%) | 46 (71%) | 0.60 |
| Cancer type | | | | 0.011 |
| Breast | 1 (1%) | 0 (0%) | 1 (2%) |  |
| GI | 5 (3%) | 4 (4%) | 1 (2%) |  |
| GYN | 1 (1%) | 1 (1%) | 0 (0%) |  |
| Head and neck | 13 (8%) | 13 (13%) | 0 (0%) |  |
| Hepatobiliary | 32 (19%) | 21 (21%) | 11 (17%) |  |
| Lung | 93 (56%) | 46 (45%) | 47 (72%) |  |
| Pancreatic | 2 (1%) | 2 (2%) | 0 (0%) |  |
| Renal | 17 (10%) | 13 (13%) | 4 (6%) |  |
| Skin | 3 (2%) | 2 (2%) | 1 (2%) |  |
| Surgery | 61 (37%) | 38 (37%) | 23 (35%) | 0.81 |
| Comorbidities | | | | |
| Hypertension | 122 (73%) | 79 (77%) | 43 (66%) | 0.11 |
| Diabetes | 57 (34%) | 42 (41%) | 15 (23%) | 0.016 |
| Hyperlipidemia | 57 (34%) | 39 (38%) | 18 (28%) | 0.16 |
| CKD | 24 (14%) | 18 (18%) | 6 (9%) | 0.13 |
| Heart Failure | 15 (9%) | 7 (7%) | 8 (12%) | 0.23 |
| Ischemic Heart Disease | 34 (20%) | 19 (19%) | 15 (23%) | 0.49 |
| Arrhythmia | 20 (12%) | 10 (10%) | 10 (15%) | 0.28 |
| Cardiovascular drugs |  |  |  |  |
| Beta-blockers | 90 (54%) | 59 (58%) | 31 (48%) | 0.20 |
| Statin | 28 (17%) | 20 (10%) | 8 (12%) | 0.22 |
| Aspirin | 31 (19%) | 19 (19%) | 12 (18%) | 0.98 |
| Calcium channel blockers | 118 (71%) | 73 (72%) | 45 (69%) | 0.75 |
| Cancer treatment | | | | |
| Anthracyclines | 13 (8%) | 7 (7%) | 6 (9%) | 0.58 |
| TKI | 59 (35%) | 36 (35%) | 23 (35%) | 0.99 |
| Vinca alkaloids | 12 (7%) | 8 (8%) | 4 (6%) | 0.68 |
| Taxanes | 25 (15%) | 21 (21%) | 4 (6%) | 0.011 |
| TOP-I inhibitor | 4 (2%) | 4 (4%) | 0 (0%) | 0.11 |
| Antimetabolites | 72 (43%) | 43 (42%) | 29 (45%) | 0.75 |
| Alkylating agents | 1 (1%) | 1 (1%) | 0 (0%) | 0.42 |
| VEGF inhibitors | 56 (34%) | 22 (20%) | 34 (52%) | <0.001 |
| Platinum | 61 (37%) | 38 (37%) | 23 (35%) | 0.81 |
| ICI types |  |  |  |  |
| PD1 inhibitors | 130 (78%) | 83 (81%) | 47 (72%) | 0.17 |
| PD-L1 inhibitors | 60 (36%) | 27 (26%) | 33 (51%) | 0.001 |
| CTLA-4 inhibitors | 2 (1%) | 1 (1%) | 1 (2%) | 0.75 |
| Combination ICIs | 3 (2%) | 2 (2%) | 1 (2%) | 0.84 |

Abbreviations: PPARγ, Peroxisome proliferator-activated receptors gamma; CKD, Chronic Kidney Disease; CTLA-4, cytotoxic T-lymphocyte-associated protein 4; ECOG-PS, Eastern Cooperative Oncology Group Performance Status; GI, gastrointestinal; GYN, gynecologic; ICI, Immune checkpoint inhibitor; PD1, programmed cell death protein 1; PD-L1, programmed death-ligand 1; TKI, tyrosine kinase inhibitor; TOP-I, Topoisomerase-inhibitor; VEGF, vascular endothelial growth factor

Supplemental table 4. Clinical benefit rate of PPAR-γ-activating vs non- PPAR-γ-activating ARB

| Group | Clinical benefit rate, n (%) | | |
| --- | --- | --- | --- |
|  | CR, PR, or SD | PD | p-value |
| PPAR-γ-user | 53 (82%) | 12 (18%) | 0.005 |
| Non-user | 62 (61%) | 40 (39%) |  |

Abbreviations: ARB, angiotensin receptor blocker; PPAR-γ, peroxisome proliferator-activated receptor-γ; CR, complete remission; PD, disease progression; PR, partial remission; SD, stable disease

Supplemental Table 5. Cox proportional hazard analysis of use of PPAR-γ and all-cause mortality and disease progression in lung cancer patients

| Analysis | Outcome | Univariate HR  (95 % IC) | P-value | Multivariate HR  (95% IC) ^a^ | P-value |
| --- | --- | --- | --- | --- | --- |
| PPAR-γ-user vs. Non-user | All-cause mortality | 0.42 (0.21-0.85) | 0.016 | 0.26 (0.09-0.77) | 0.016 |
|  | Disease progression or mortality | 0.50 (0.30-0.84) | 0.009 | 0.40 (0.19-0.84) | 0.015 |

Abbreviations: PPAR-y, peroxisome proliferator-activated receptor-γ

^a^ Multivariate analysis includes variables: age, sex, cancer type, cancer stage, Eastern Cooperative Oncology Group Performance Status, surgery, underlying comorbidities such as hypertension, diabetes mellitus, chronic kidney disease, heart failure, ischemic heart disease, arrhythmia, hyperlipidemia, the use of medications such as beta-blocker, calcium channel blocker, statin, aspirin, cancer treatment including VEGF inhibitor, taxanes, vinca alkaloids, platinums, tyrosine kinase inhibitors, anthracyclines alkylating agents, topoisomerase-1 inhibitors, antimetabolites, ICI class including PD-1 inhibitors, PD-L1 inhibitors, CTLA-4 inhibitors, and combination immune checkpoint inhibitor therapy
